# Assessment of Liver Function for Evaluation of Long-Term Outcomes of Intrahepatic Cholangiocarcinoma: A Multi-Institutional Analysis of 620 Patients

**DOI:** 10.3389/fonc.2020.00525

**Published:** 2020-04-28

**Authors:** Hui Li, Jiaxin Li, Jinju Wang, Hailing Liu, Bole Cai, Genshu Wang, Hong Wu

**Affiliations:** ^1^Department of Liver Surgery, Liver Transplantation Division, West China Hospital, Sichuan University, Chengdu, China; ^2^Laboratory of Liver Surgery, West China Hospital, Sichuan University, Chengdu, China; ^3^Department of Hepatic Surgery and Liver Transplantation Center, The Third Affiliated Hospital of Sun Yat-sen University, Guangzhou, China

**Keywords:** intrahepatic cholangiocarcinoma, albumin–bilirubin grade, albumin-to-alkaline phosphatase ratio, surgery, prognostic utility

## Abstract

**Background:** Liver function is a routine laboratory test prior to curative liver resection. It remains unclear whether the albumin–bilirubin (ALBI) grade and albumin-to-alkaline phosphatase ratio (AAPR) can predict long-term outcomes of surgically treated patients with intrahepatic cholangiocarcinoma (ICC).

**Methods:** This study investigated the correlation between ALBI grade and AAPR with overall survival (OS) after liver resection and then compared their accuracy to the Child–Pugh score. Harrell's concordance index (C-index) and Akaike information criterion (AIC) were used to compare accuracy of models.

**Results:** A total of 620 ICC patients were included, 477 in derivation cohort and 143 for validation. 0.348 was identified as the cutoff value for AAPR after calculating the Youden index. In the derivation cohort, elevated ALBI grade was associated with worse prognosis [hazard ratio (HR): 1.751, 95% confidence interval (CI): 1.329 to 2.306], and a decreased AAPR value was correlated with shorter OS (HR: 1.969, 95% CI: 1.552 to 2.497). Multivariate analysis suggested that the ALBI grade, AAPR, CA19-9, tumor number, and microvascular invasion were independent prognostic predictors for OS. ALBI grade and AAPR showed more accuracy in evaluating OS for surgically treated ICC patients than the Child–Pugh score (C-index: 0.559, 0.600 vs. 0.528; AIC: 3023.84, 3007.73 vs. 3034.66). Our findings were validated in an independent cohort from another clinical center.

**Conclusions:** Importantly, the ALBI grade and AAPR showed greater discriminatory power than the Child–Pugh score in assessing long-term outcomes following hepatectomy for ICC. The AAPR was more accurate than the ALBI grade. It was beneficial to consider the ALBI grade and AAPR as useful surrogate markers to identify patients at risk of poor postoperative outcomes.

## Introduction

Liver cancer remains the sixth most commonly diagnosed cancer as well as the fourth leading cause of malignancy-related mortality worldwide, which gives rise to nearly 841,000 new cases and 782,000 deaths every year (1). Intrahepatic cholangiocarcinoma (ICC) is one of the subtypes of cholangiocarcinoma, which accounts for about 10–15% of all primary hepatic malignancies1 (2, 3). The incidence and mortality of ICC rank only behind hepatocellular carcinoma (HCC) and increase aggressively in recent years (4). In contrast to HCC, ICC is a cancer associated with rapid progression and a dismal 5-year survival rate <5–10% (5). Surgery remains the most efficient treatment for patients with early-stage tumor (6). However, prognosis after hepatic resection is still unsatisfactory with a 5-year survival rate <40% for ICC patients with R0 resection (6, 7). Systematic chemotherapy based on gemcitabine and cisplatin has become standard strategy for advanced or metastasis ICC, but the median survival time is <1 year (6). Therefore, it is necessary to explore useful prognostic factors that facilitate the selection of appropriate surgical candidates and therapeutic strategies.

The 7th edition of the American Joint Committee on Cancer (AJCC) Staging Manual introduced a new staging system, which has become the most commonly used for ICC (8, 9). In the current 8th edition, the T1 category has been subdivided to T1a and T1b according to a maximum tumor diameter of 5 cm (10). Wang et al. proposed a prognostic nomogram, comprising serum carcinoembryonic antigen (CEA), CA 19-9, tumor number and diameter, vascular invasion, lymph node, and local extrahepatic metastasis, for ICC that underwent partial hepatectomy (3). Apart from tumor staging systems, serum parameters and prognostic models are explored in the assessment of prognosis of ICC. Tumor markers, such as CEA and CA19-9, have been confirmed for their value in predicting prognosis of ICC patients (11, 12). Inflammation-based prognostic models such as neutrophil-to-lymphocyte ratio (NLR) (13), platelet-to-lymphocyte ratio (PLR) (14), and lymphocyte-to-monocyte ratio (LMR) (13) have been introduced to evaluate prognosis. Moreover, the underlying liver function also plays an important role in prognostic outcomes of ICC patients. Generally, the Child–Pugh grade is used to evaluate hepatic function reserve prior to hepatectomy. It has been reported to be an independent prognostic factor for overall survival (OS) for ICC (15). Recently, the albumin–bilirubin (ALBI) grade and albumin-to-alkaline phosphatase ratio (AAPR) were introduced to assess liver function. Several studies have investigated their prognostic value in HCC, demonstrating that decreased AAPR and elevated ALBI grade were associated with worse prognosis after hepatic resection (16–18). However, their application value in predicting prognosis of ICC patients remains unclear.

In the present study, we aimed to assess the prognostic significance of scoring systems based on preoperative liver function for surgically treated ICC and then propose simple and feasible models to stratify patients at different risk of postoperative outcomes.

## Methods

### Study Population

In this multicenter retrospective study, a total of 620 surgically treated ICC patients at West China Hospital and the Third Affiliated Hospital of Sun Yat-sen University were consecutively included. Patient set from the West China Hospital between January 2009 and December 2017 were used to explore our initial practice (derivation cohort) and then the findings were verified in an independent cohort from the Third Affiliated Hospital of Sun Yat-sen University (located in southern China, validation cohort). All the enrolled patients were newly pathologically diagnosed ICC and underwent surgical resection with curative intent for first time. The exclusion criteria were as follows: patients received radiofrequency ablation, transarterial chemoembolization, chemotherapy, or other anti-cancer therapies before hepatectomy; patients with extrahepatic metastasis; patients who underwent surgical resection for tumor rupture; and those without complete medical records. All eligible patients or their relative provided written informed consent. This study was approved by the Ethics Committee of the West China Hospital and the Third Affiliated Hospital of Sun Yat-sen University, in accordance with the guidelines of the 1975 Declaration of Helsinki (19).

### Clinical Data Collection and Follow-Up

All the patient information and preoperative laboratory parameters were reviewed and retrieved from the hospital electronic or handwritten medical records. The ALBI was calculated by the formula (log_10_bilirubin × 0.66)–(albumin × 0.085), where bilirubin was in mol/L and albumin was in g/L (20). Patients were classified according to the previously reported cutoffs ≤ -2.60 (ALBI grade 1), >-2.60 to ≤ -1.39 (ALBI grade 2), and ≥-1.39 (ALBI grade 3). AAPR was calculated from dividing the ALB level by serum ALP level, where ALB was in g/L and ALP was in U/L (16). Patients were also stratified according to the cutoffs. Moreover, tumor-related clinicopathological characteristics including differentiation, number of tumor nodules, maximum tumor diameter, macrovascular invasion, microvascular invasion (MVI), and extrahepatic metastasis were also acquired. MVI was defined as histologically identified vascular invasion of small vessels, while macrovascular invasion was defined as radiologically diagnosed vascular invasion of large vessels or macroscopic thrombosis. The TNM stages were stratified according to the 8th edition of the AJCC Staging Manual. Patients were followed up according to National Comprehensive Cancer Network (NCCN), regularly contrast-enhanced ultrasonography per month at first year, and then every 3 months for 2 years, and then every 6 months thereafter. Besides, we contact those who determined not to go back to the hospital to reexamination through telephone follow-up survey. Few patients (seven in West China Hospital and four in the Third Affiliated Hospital of Sun Yat-sen University) lost to follow-up at the end of follow-up in December 2018. Additionally, five patients (four in West China Hospital and one in the Third Affiliated Hospital of Sun Yat-sen University) were excluded due to incomplete medical records (three without intraoperative outcomes and two without preoperative liver function test).

### Statistical Analysis

The statistical analyses were performed by using the software of SPSS (version 22.0, Chicago, IL, USA), MedCalc (version 15.2.2.0, Ostend, Belgien), and GraphPad Prism (version 8.0, San Diego, California, USA). Harrell's concordance index (C-index) and Akaike information criterion (AIC) were calculated by using R (https://www.r-project.org/, v3.3.4). Receiver-operating characteristic (ROC) curves were applied to determine the optimal cutoff value as the Youden index attained maximum value. Pearson's chi square test and Student's *t*-test were used to investigate the correlation of categorical and continuous variables to AAPR level, respectively. Kaplan–Meier curves were plotted for derivation and validation cohort according to each cutoff value, and their differences were tested using log-rank test. Those clinicopathological parameters with *P* < 0.2 in the univariable Cox proportional hazards regression were considered for generating multivariable Cox regression (enter method) to identify potential independent prognostic factors for OS (21). A two-tailed *P* < 0.05 was considered statistically significant.

## Results

### Derivation Cohort

In the derivation cohort, 477 patients [227 (47.6%) male; median (IQR) age, 58 (49.5, 64) years] were finally recruited after excluding the only one with preoperative liver function of ALBI grade 3. Table 1 summarized baseline characteristics of the patient cohorts. No patient was stratified into Child–Pugh grade C; 7 (0.8%) were Child–Pugh B. Among patients with Child–Pugh A, 407 (85.3%) were 5 points, whereas 66 (13.8%) were 6 points. A cutoff value of 0.348 for AAPR was identified by using ROC. A total of 292 (61.2%) patients with AAPR value more than 0.348 were classified into elevated AAPR group (AAPR grade 1), whereas 185 (38.8%) were stratified into decreased group (AAPR grade 2). By using ALBI grade, 387 (81.1%) patients were stratified into ALBI grade 1 and 90 (18.9%) were stratified in ALBI grade 2. Eighty-three (17.4%) patients also suffered from hepatolithiasis. The carbohydrate antigen 19-9 (CA19-9) was available for 467 patients. Since the reference range was 22 U/ml, elevated serum CA19-9 was detected in 69.2% of the patients.

**Table 1:** Baseline characteristics of patients.

**Variables**	**Derivation cohort** **(*n* = 477)**	**Validation cohort** **(*n* = 143)**
**Patient factors/Laboratory parameters**		
Age [year, median (IQR)]	58 (49.5–64)	59 (51–67)
Male gender, *n* (%)	227 (47.6)	83 (58)
HBsAg [positive, *n* (%)]	139 (29.1)	35 (24.6)
HCV, *n* (%)	2 (0.4)	2 (1.4)
Hepatolithiasis, *n* (%)	83 (17.4)	12 (8.4)
Child–Pugh score, *n* (%)		
5	407 (85.3)	125 (87.4)
6	66 (13.8)	15 (10.5)
7	7 (0.4)	3 (2.1)
Ascites, *n* (%)	47 (9.9)	5 (3.5)
ALB [g/L, mean (SD)]	42.7 (4.6)	41.7 (4.1)
TBIL [μmol/L, mean (SD)]	14.3 (8.5)	14.2 (7.7)
ALP [U/L, mean (SD)]	136.6 (95.1)	135.3 (76.1)
ALBI grade, *n* (%)		
1	387 (81.1)	103 (72)
2	90 (18.9)	40 (28)
AAPR grade, *n* (%)		
1 (>0.348)	293 (61.4)	76 (53.1)
2 (≤0.348)	184 (38.6)	67 (46.9)
CA19-9		
<22, *n* (%)	137 (28.7)	37 (25.9)
≥22, *n* (%)	330 (69.2)	105 (73.4)
Not available	10 (2.1)	1 (0.7)
**Histological and gross features of tumors**		
Tumor size [cm, mean (SD)]	6 (2.7)	6.1 (2.4)
Solitary tumor, *n* (%)	337 (70.6)	88 (61.5)
Tumor differentiation	17/55	10/67
Well	12 (2.5)	5 (3.5)
Moderate	362 (75.9)	113 (79)
Poor	78 (16.4)	22 (15.4)
Not available	25 (5.2)	3 (2.1)
Macrovascular invasion, *n* (%)	106 (22.2)	52 (36.4)
Microvascular invasion, *n* (%)	51 (10.7)	14 (9.8)
Cirrhosis, *n* (%)	136 (28.5)	20 (14)
**Prognostic outcome**		
Overall survival, months, mean (95% CI)	24.9 (23.0, 26.8)	30.5 (26.7, 34.2)

*ALBI, albumin–bilirubin; AAPR, albumin-to-alkaline phosphatase ratio; ALB, albumin; TBIL, total bilirubin; ALP, alkaline phosphatase; CA19-9, carbohydrate antigen 19-9; IQR, interquartile range; SD, standard deviation; CI, confidence interval*.

**Figure 1:**
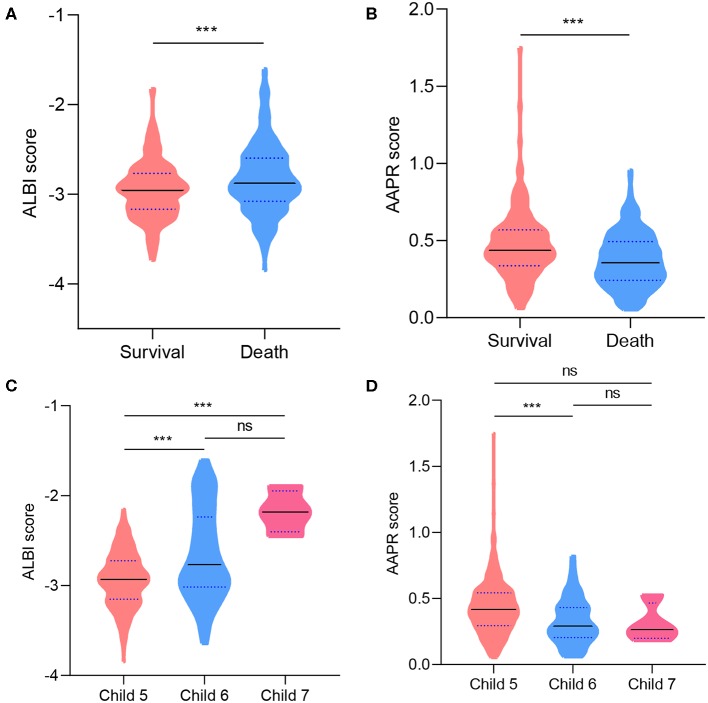
Violin plots showing the distribution of ALBI and AAPR score: in survival and death group at the end of follow-up **(A,B)**; for patients with different Child–Pugh scores **(C,D)**. Solid lines represent median value; dotted lines represent quartiles. *P < 0.05; **P < 0.01; ***P < 0.001; ns, not significant.

**Figure 2:**
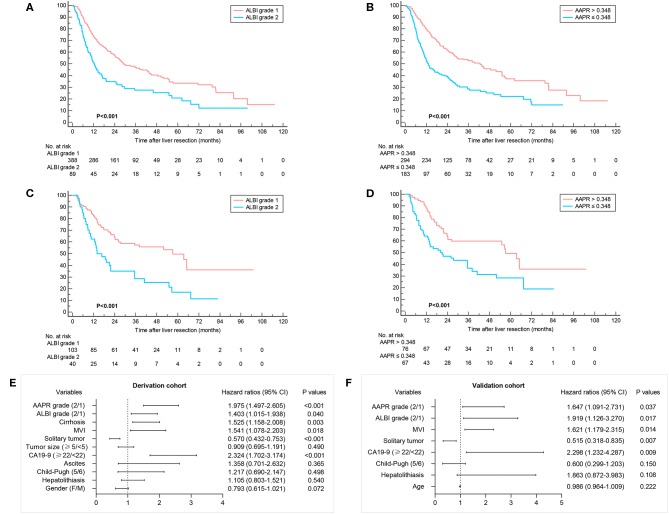
Overall survival according to groups defined by the ALBI grade and cutoff value of AAPR. Kaplan–Meier curves of 477 ICC patients in the derivation cohort **(A,B)** and 143 patients in the validation cohort **(C,D)**. Multivariate analyses showing independent prognostic factors in derivation and validation cohort **(E,F)**.

The correlation between patient characteristics with ALBI and AAPR were summarized in Table 2. A higher ratio of Child–Pugh score 5 and larger tumor size were observed in ALBI grade 1 and AAPR grade 1. The mean OS was 24.9 months, 203 (42.6%) survived at the end of follow-up, whereas 274 (57.4%) died. A comparison of ALBI and AAPR values between survival and death demonstrated a decreased AAPR value and increased ALBI value in the dead group (Figures 1A,B). In patients with Child–Pugh A, an elevated AAPR value and decreased ALBI were observed in patients with Child–Pugh score 5 (Figures 1C,D). Kaplan–Meier analyses with log-rank test suggested that ALBI grade 1, elevated AAPR value, normal serum CA19-9 level, and solitary tumor correlated with better prognosis, whereas patients with microvascular invasion, cirrhosis, poor tumor differentiation, and Child–Pugh score 6 were related to poor survival outcomes (Figures 2A,B and Supplementary Figure 1). Among 19 clinicopathological characteristics, 14 were identified as potentially relevant with *P* < 0.2 in the univariate Cox regression analyses. Twelve of them were introduced in consequent multivariate Cox proportional hazards regression model, and 6 were identified as independent prognostic predictors (Figure 2E and Supplementary Table 2).

**Table 2:** Correlation of characteristics with the ALBI and AAPR of 477 ICC patients treated with surgical resection in the derivation cohort.

**Characteristics**	**ALBI**			**AAPR**		
	**Grade 1 (*n* = 387)**	**Grade 2 (*n* = 90)**	***P* value**	**>0.348 (*n* = 293)**	**≤0.348 (*n* = 184)**	***P*-value**
Age [year, mean (SD)]	57.1 (10.7)	56.2 (10.9)	0.958	56.9 (10.9)	56.9 (10.4)	0.998
Male gender, *n* (%)	175 (45.2)	52 (57.8)	0.032	143 (48.8)	84 (45.7)	0.511
HBsAg [positive, *n* (%)]	106 (27.5)	33 (37.1)	0.091	106 (36.6)	33 (18.0)	<0.001
Hepatolithiasis, *n* (%)	61 (15.8)	22 (24.4)	0.061	40 (13.7)	43 (23.4)	0.006
Child–Pugh score, *n* (%)						
5	348 (89.9)	59 (65.5)	<0.001	265 (90.4)	142 (77.2)	<0.001
6	39 (10.1)	27 (30.0)		27 (9.2)	39 (21.2)	
Ascites, *n* (%)	36 (9.3%)	11 (12.2)	0.432	24 (8.2)	23 (12.5)	0.152
ALB [g/L, mean (SD)]	43.9 (2.9)	36.6 (3.1)	<0.001	43.5 (3.5)	41.2 (4.6)	<0.001
TBIL [μmol/L, mean (SD)]	13.6 (5.7)	17.1 (15.4)	0.001	13.8 (5.8)	15.1 (11.6)	0.102
ALP [U/L, mean (SD)]	128.1 (85.1)	172.7 (123.8)	<0.001	88.7 (23.1)	212.6 (114.8)	<0.001
CA19-9						
<22, *n* (%)	114 (30.2)	23 (25.8)	0.439	93 (32.4)	44 (24.4)	0.075
≥22, *n* (%)	264 (69.8)	66 (74.2)		194 (67.6)	136 (75.6)	
Tumor size [cm, mean (SD)]	5.8 (2.5)	6.6 (3.3)	0.014	5.5 (2.3)	6.8 (3.2)	<0.001
Solitary tumor, *n* (%)	276 (71.3)	61 (67.8)	0.536	217 (74.1)	120 (65.2)	0.113
Tumor differentiation
Well	10 (2.6)	2 (2.2)	0.068	10 (3.4)	2 (1.1)	0.022
Moderate	302 (78.0)	60 (66.7)		219 (74.7)	143 (77.7)	
Poor	55 (14.2)	23 (25.6)		54 (18.4)	24 (13)	
Macrovascular invasion, *n* (%)	82 (21.2)	24 (26.7)	0.324	50 (17.1)	56 (30.4)	0.001
Microvascular invasion, *n* (%)	43 (11.1)	8 (8.9)	0.579	29 (9.9)	22 (12.0)	0.550
Cirrhosis, *n* (%)	102 (26.4)	34 (37.8)	0.038	90 (30.7)	46 (25)	0.211
Overall survival, months, mean (95% CI)	25.6 (23.5, 27.6)	21.9 (16.9, 26.9)		27.4 (24.9, 29.8)	20.9 (18.1, 23.9)	

*ALBI, albumin–bilirubin; AAPR, albumin-to-alkaline phosphatase ratio; ALB, albumin; TBIL, total bilirubin; ALP, alkaline phosphatase; CA19-9, carbohydrate antigen 19-9; IQR, interquartile range; SD, standard deviation; CI, confidence interval*.

**Figure 3:**
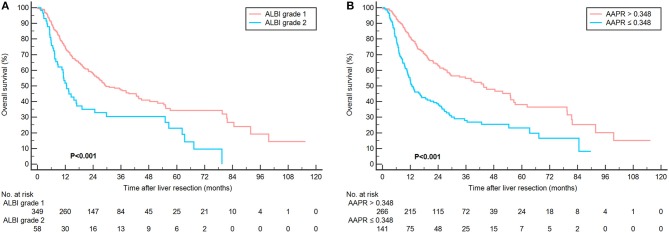
Kaplan-Meier curves showing overall survival according to ALBI **(A)** and AAPR grade **(B)** in 407 patients with Child-Pugh score 5.

In the subgroup analysis of patients with Child–Pugh score 5, the prognostic outcome was further analyzed according to ALBI and AAPR grades. Three hundred forty-nine (85.7%) were stratified as ALBI grade 1 and 266 (65.4%) were classified into AAPR grade 1. Consistently, better prognosis was also found in patients with ALBI grade 1 and AAPR value more than 0.348 (Figure 3).

C-index and AICs were also compared between ALBI grade and AAPR with Child–Pugh score in predicting survival outcomes. The results suggested that the ALBI grade and AAPR were more representative in evaluating OS for surgically treated ICC patients than Child–Pugh score (C-index: 0.559, 0.600 vs. 0.528; AIC: 3023.84, 3007.73 vs. 3034.66) (Table 3). Moreover, the AAPR showed more accurate predicting value than the ALBI grade.

**Table 3:** Comparisons of the value of three models in predicting prognosis of overall survival among the patients in derivation and validation cohort.

**Models**	**C-index**	**95% CI**	***P*-value**	**AIC**
**Derivation cohort**
Child–Pugh score	0.528	0.504–0.553	Ref	3034.66
ALBI grade	0.559	0.532–0.586	0.023	3023.84
AAPR grade	0.601	0.570–0.632	< 0.001	3007.73
**Validation cohort**				
Child–Pugh score	0.533	0.489–0.705	Ref	710.09
ALBI grade	0.585	0.531–0.639	0.039	702.20
AAPR grade	0.600	0.545–0.655	0.012	700.61

*ALBI, albumin–bilirubin; AAPR, albumin-to-alkaline phosphatase ratio; CI, confidence interval; Ref, reference; AIC, Akaike information criterion. A lower AIC, indicating lower loss of information, was more representative of the model*.

In addition, we also compared perioperative outcomes between ICC patients in different ALBI and AAPR groups. The results showed similar operative time, estimated blood loss, blood transfusion, postoperative complications, and hospitalization between ICC patients with different preoperative ALBI score and AAPR levels (Supplementary Table 4). There was a tendency that patients with elevated AAPR levels correlated with decreased frequency of postoperative complications with a *P* < 0.1.

### Validation Cohort

Consecutive ICC patients treated with surgical resection at the Third Affiliated Hospital of Sun Yat-sen University from January 2011 to December 2017 were recruited to validate our findings. After the exclusion of one patient with preoperative ALBI grade 3, 143 patients [83 (58%) male, median (IQR) age, 59 (51, 67) years] were included. The correlation of tumor characteristics with ALBI grade and AAPR grade is shown in Supplementary Table 1. Consistent with derivation cohort, patients in ALBI grade 1 and AAPR grade 1 correlated with an increased ratio of Child–Pugh score 5 and mean OS.

A total of 125 patients (87.4%) were associated with Child–Pugh score 5. By calculating ALBI score and AAPR values, 103 (72%) were stratified into ALBI grade 1 and 76 (53.1%) patients have an AAPR value more than 0.348. The mean OS was 30.5 months. In the univariate analyses, Child–Pugh score, ALP, ALBI grade, AAPR, CA19-9, tumor number, and microvascular invasion were possible variables that correlated with patients' prognosis (Figures 2C,D). The ALBI grade, AAPR, CA19-9, tumor number, and microvascular invasion were identified as independent prognostic predictors for postoperative survival (Figure 2F and Supplementary Table 2). The subgroup analyses of ICC patients in the validation cohort are shown in Supplementary Figure 2.

The short-term outcomes were also comparable between 143 ICC patients in different ALBI and AAPR groups from the Third Affiliated Hospital of Sun Yat-sen University (Supplementary Table 4).

### Discussion

In the present study, we evaluated the potential impact of scoring systems based on preoperative liver function on prognostic outcomes in surgically treated ICC. Child–Pugh score and ALBI and AAPR grade showed their prognostic value in predicting OS. The multivariate analysis identified key variables that influenced OS, including tumor features (CA19-9, tumor number, and microvascular invasion) and liver function (ALBI and AAPR grade). These results were in accordance to previous studies (22).

In contrast to tumor characteristics, the underlying liver function is routinely detected prior to operation. As is well-known, preoperative liver function has an impact on postoperative outcomes for patients undergoing curative resection. Child–Pugh score is one of the most widely used for assessing liver function. It is also an indication for selecting proper candidate for surgical treatments. However, there are several limitations, including arbitrary cutoff points for laboratory indices and subjective clinical assessment of ascites and hepatic encephalopathy (23, 24). The ALBI grade, proposed by Johnson and colleagues, calculated from albumin and bilirubin, has been demonstrated to be a useful prognostic predictor for patients undergoing liver resection (17, 25). Serum ALB, a kind of protein synthesized in liver, is an indicator that reflects the protein synthetic capability. Moreover, it remains a modulator for inflammatory response, which is essential in retardation of liver cancer (26). In the univariate analyses, ALB was a potential factor relating to OS. However, as a component of ALBI, it had to be excluded in multivariate analysis. Our results were consistent with previous studies, validated the accurate prognostic value of ALBI grade for surgically treated ICC patients. Recently, Tsilimigras et al. demonstrated that the ALBI score was associated with both short- and long-term outcomes following hepatectomy for ICC and could prove a useful surrogate marker to identify patients at risk for adverse outcomes (27). However, in our study, we found that the perioperative outcomes were comparable between different ALBI and AAPR grades in both derivation and validation cohort.

Initially, only ALB, ALP, and TBIL were put into multivariate analysis (Supplementary Table 3). The results showed that ALB and ALP were independent prognostic factors, whereas TBIL was not. Thus, we combined ALB and ALP to create a stronger prognostic factor-AAPR and compared its accuracy to ALBI and Child–Pugh classification. The AAPR grade based on a cutoff value of 0.348 was also an independent prognostic predictor for OS. Calculated from dividing the ALB by ALP, it was introduced by Chan et al. initially to predict prognosis of HCC patients who underwent surgical resection and palliative therapy (16). Cai et al. validated the prognostic value of AAPR in advanced HCC patients who did not receive any standard anti-cancer treatments (28). The serum ALP, another one of the most routinely detected parameters in laboratory test, is a hydrolase enzyme that contains a mixture of ALP isoenzymes from liver, bones, kidney, and placenta. Several studies have reported that the ALP level increases during childhood and other diseases such as hepatic diseases, osteomalacia, and bone tumor (29, 30). Patients with biliary diseases are always associated with elevated serum ALP level. Previous studies have reported that ALP was an independent prognostic factor for ICC patients (31, 32). Zhang et al. have evaluated the prognostic effect of serum liver enzymes in ICC patients and demonstrated that both ALB and ALP were predictive factors (31). However, the enrolled 173 patients were associated with locally advanced or metastatic tumors. In our study, 620 patients from two centers were associated with early-stage ICC and underwent curative resection, which remained the most efficient treatment for ICC patients. We also examined the prognostic significance of ALB and ALP either alone or in combination, and found that ALBI and AAPR showed a promising effect on stratifying ICC patients into a different risk of postoperative outcomes. They might show a stronger prognostic significance than ALB, ALP, and TBIL alone. Our results did not conflict with these studies, showed ALP as a potential relevant in the univariate analysis. It was also excluded in multivariable analysis. Our results also identified the decreased AAPR score as an independent risk factor of postoperative survival for surgically treated ICC patients.

Importantly, our present analysis suggested that the ALBI grade and AAPR score exhibited a greater discriminatory power than Child–Pugh score. Previous studies demonstrated at least equal discriminatory power, in prognostic terms, of ALBI score to Child–Pugh score (33). Apart from containing two subjective parameters (encephalopathy and ascites), Child–Pugh score is advocated for evaluating liver function with the precondition that patients suffered from cirrhosis. However, only a few ICC patients had cirrhosis, with a proportion of 25.2% in our current study. Therefore, it was not a surprise that the Child–Pugh score was not found to be an independent factor with respect to postoperative survival. To compare the accuracy of these three models, subgroup analysis was performed to investigate the performance of AAPR and ALBI grade within the Child–Pugh score 5. The result showed distinct prognosis for patients with different AAPR and ALBI grade, indicating their potential advantages over Child–Pugh classification. Indeed, the degree of liver functional reserve varied greatly among patients with same Child–Pugh score. For patients with severe cirrhosis, their Child–Pugh score might be the same as those with very early stage tumors. Consistent with previous studies, ALBI grade exhibited a greater discriminatory power than Child–Pugh score (33–35). Furthermore, consistent with ALBI grade, AAPR score showed a greater stratifying ability in representing liver function reserve for ICC patients treated with surgical resection.

ALBI and AAPR are easily accessible laboratory tests that are routinely detected for patients with liver diseases. Additionally, unlike other serodiagnosis or iconographical detections, they are low-cost tests that will not increase the total medical costs. However, it should be noted that the ALBI and AAPR scores are not tumor staging systems since they only measure preoperative liver function, taking no account of tumor-related factors. Further models combining ALBI, AAPR, and other tumor characteristics (tumor size, number, microvascular invasion status, and CA19-9) are in need to evaluate prognostic outcomes for surgically treated ICC patients.

There were several limitations that warrant consideration when interpreting our findings. Firstly, although our initial findings were validated by an independent cohort from different areas inside our country, it was limited by its retrospective design. Secondly, the etiologies for ICC differed from different geographic regions (especially Asia with Europe and America); further prospective international multicenter studies were required to verify our primary findings. In addition, in these two centers, to ensure patients' safety, part of patients received surgical resection after treating with hepatic protectants. Hence, few patients with Child–Pugh grade B were included. Additionally, the mechanisms underlying why liver function impact survival outcomes were not analyzed in the present study. In the future, we will perform a basic research to investigate the possible mechanisms using animal models and *in vitro* cell experiments. Finally, owing to insufficient ward beds, prompt surgical treatments were not available for a subset of ICC patients with operative indication; the referral bias could not be completely avoided.

## Conclusions

The present study provided compelling evidence that the AAPR score and ALBI grade were validated prognostic factors for surgically treated ICC patients. They enabled dividing these patients into independent groups before surgical treatment. Since three widely available laboratory parameters were involved, the AAPR score and ALBI grade were more objective, low-cost, and readily available without special tests. Moreover, the preoperative ALBI grade and AAPR score showed greater discriminatory power than Child–Pugh grade. The AAPR was more accurate than ALBI grade. Even among patients with Child–Pugh score 5, they also exhibited their accurate prognostic value in predicting postoperative survival outcome. It was beneficial to consider the ALBI grade and AAPR as useful surrogate markers to identify patients at risk of poor postoperative outcomes, and future prospective validation is required.

## Data Availability Statement

The datasets generated for this study are available on request to the corresponding author.

## Ethics Statement

The studies involving human participants were reviewed and approved by the ethics committee of the West China Hospital and the Third Affiliated Hospital of Sun Yat-sen University. The patients/participants provided their written informed consent to participate in this study.

## Author Contributions

HLi, HW, and GW: conceptualization. HLi, JL, and JW: data curation. HLi, BC, and HLiu: formal analysis. GW and HW: supervision. HLi, JL, and JW: writing—original draft. All the authors approved the final version of manuscript.

## Conflict of Interest

The authors declare that the research was conducted in the absence of any commercial or financial relationships that could be construed as a potential conflict of interest.
