# Supplementary figures and images for: Assessment of Liver Function for Evaluation of Long-Term Outcomes of Intrahepatic Cholangiocarcinoma: A Multi-Institutional Analysis of 620 Patients
Source: Front Oncol. 2020 Apr 28;10:525. doi: 10.3389/fonc.2020.00525 (PMC7198721; doi:10.3389/fonc.2020.00525)

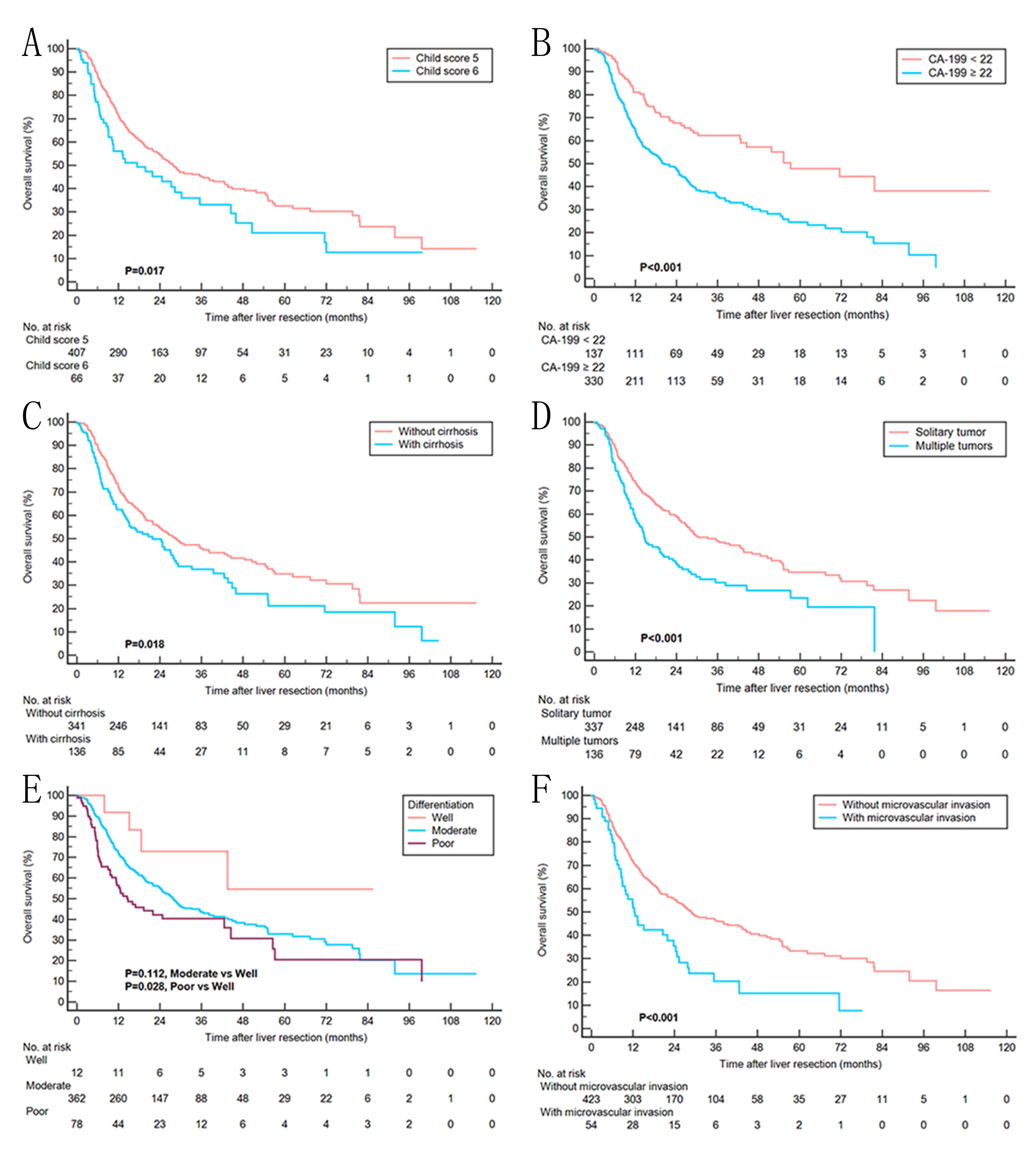

Supplement: Supplementary file 1 [file Image_1.jpg]
