# Supplementary material for: Assessment of Liver Function for Evaluation of Long-Term Outcomes of Intrahepatic Cholangiocarcinoma: A Multi-Institutional Analysis of 620 Patients
Source: Front Oncol. 2020 Apr 28;10:525. doi: 10.3389/fonc.2020.00525 (PMC7198721; doi:10.3389/fonc.2020.00525)
Supplement: Supplementary file 2 [file Table_1.DOCX]

Supplementary table 1. Correlation of characteristics with the ALBI and AAPR of 143 ICC patients treated with surgical resection in the validation cohort.

| **Characteristics** | **ALBI** | | | **AAPR** | | |
| --- | --- | --- | --- | --- | --- | --- |
|  | Grade 1 (*n*=103) | Grade 2 (*n*=40) | P value | >0.348 (n=76) | ≤0.348 (n=67) | P value |
| **Age [year, mean (SD)]** | 57.1 (10.7) | 56.2 (10.9) | 0.958 | 59.6 (9.8) | 56.2 (11.5) | 0.77 |
| **Male gender, n (%)** | 59 (57.3) | 24 (60.0) | 0.851 | 37 (48.7) | 46 (68.7) | 0.018 |
| **HBsAg, [positive, n (%)]** | 27 (36.2) | 8 (20.5) | 0.528 | 20 (26.3) | 15 (22.7) | 0.691 |
| **Hepatolithiasis, n (%)** | 10 (9.7) | 2 (5.0) | 0.516 | 5 (6.6) | 7 (10.4) | 0.541 |
| **Child-Pugh score, n (%)** |  |  |  |  |  |  |
| **5** | 99 (96.1) | 26 (65.0) | <0.001 | 74 (97.4) | 51 (76.1) | <0.001 |
| **6** | 4 (3.9) | 11 (27.5) |  | 2 (2.6) | 13 (19.4) |  |
| **Ascites, n (%)** | 4 (3.9) | 1 (2.5) | 1.000 | 2 (2.6) | 3 (4.5) | 0.660 |
| **ALB, [g/L, mean (SD)]** | 43.9 (2.9) | 36.6 (3.1) | <0.001 | 43.3 (2.9) | 37.3 (2.9) | <0.001 |
| **TBIL, [μmol/L, mean (SD)]** | 13.6 (5.7) | 17.1 (15.4) | 0.001 | 12.9 (4.3) | 17.9 (12.9) | 0.001 |
| **ALP, [U/L, mean (SD)]** | 128.1 (85.1) | 172.7 (123.8) | <0.001 | 122.8 (52.1) | 172.1 (115.4) | 0.001 |
| **CA19-9** |  |  |  |  |  |  |
| **<22, n (%)** | 29 (28.2) | 8 (20.5) | 0.399 | 23 (30.3) | 14 (21.2) | 0.245 |
| **≥22, n (%)** | 74 (71.8) | 31 (79.5) |  | 53 (69.7) | 52 (78.8) |  |
| **Tumor size, [cm, mean (SD)]** | 5.8 (2.5) | 6.6 (3.3) | 0.014 | 6.1 (2.3) | 6.3 (2.7) | 0.662 |
| **Solitary tumor, n (%)** | 67 (65.0) | 21 (52.5) | 0.184 | 51 (67.1) | 37 (55.2) | 0.170 |
| **Tumor differentiation** |  |  |  |  |  |  |
| **Well** | 4 (3.9) | 1 (2.5) | 0.601 | 4 (5.3) | 1 (1.5) | 0.525 |
| **Moderate** | 82 (79.6) | 31 (77.5) |  | 61 (80.3) | 52 (77.6) |  |
| **Poor** | 14 (3.9) | 8 (20.0) |  | 10 (13.2) | 12 (17.9) |  |
| **Macrovascular invasion, n (%)** | 35 (34.0) | 17 (42.5) | 0.443 | 23 (30.3) | 29 (43.3) | 0.113 |
| **Microvascular invasion, n (%)** | 9 (8.7) | 5 (12.5) | 0.536 | 6 (7.9) | 8 (11.9) | 0.570 |
| **Cirrhosis, n (%)** | 13 (12.6) | 7 (17.5) | 0.591 | 11 (14.5) | 9 (13.4) | 1.000 |
| **Overall survival, months, mean (95% CI)** | 32.6 (28.3, 37.0) | 24.9 (17.8, 32.1) |  | 35.9 (30.7, 41.2) | 24.3 (19.4, 29.3) |  |

ALBI, albumin-bilirubin; AAPR, albumin-to-alkaline phosphatase ratio; ALB, albumin; TBIL, total bilirubin; ALP, alkaline phosphatase; CA19-9, carbohydrate antigen19-9; IQR, interquartile range; SD, standard deviation; CI, confidence interval.

Supplementary table 2. Univariate analyses using ICC patients from derivation and validation cohort.

| Variables | Derivation cohort | | | Validation cohort | | |  |
| --- | --- | --- | --- | --- | --- | --- | --- |
|  | HR | 95% CI | *P* | HR | 95% CI | *P* | |
| Age | 0.998 | 0.987-1.009 | 0.775 | 0.981 | 0.960-1.002 | 0.079 |  |
| Gender (F/M) | 0.823 | 0.649-1.043 | 0.108 | 0.821 | 0.524-1.288 | 0.391 |  |
| HBsAg | 1.168 | 0.903-1.510 | 0.237 | 1.025 | 0.615-1.707 | 0.925 |  |
| Hepatolithiasis | 1.340 | 1.002-1.790 | 0.048 | 1.922 | 0.955-3.868 | 0.067 |  |
| Child-Pugh (5/6) | 0.678 | 0.492-0.934 | 0.017 | 0.424 | 0.233-0.769 | 0.005 |  |
| Ascites (+/-) | 1.309 | 0.900-1.902 | 0.159 | 1.772 | 0.431-7.282 | 0.159 |  |
| ALB | 0.952 | 0.926-0.980 | 0.001 | 0.966 | 0.914-1.021 | 0.219 |  |
| TBIL | 1.003 | 0.981-1.013 | 0.686 | 1.014 | 0.992-1.036 | 0.225 |  |
| ALP | 1.002 | 1.001-1.003 | 0.001 | 1.003 | 1.001-1.005 | 0.028 |  |
| ALBI (2/1) | 1.751 | 1.329-2.306 | <0.001 | 2.214 | 1.413-3.468 | 0.001 |  |
| AAPR (2/1) | 1.969 | 1.552-2.497 | <0.001 | 2.013 | 1.291-3.139 | 0.002 |  |
| CA19-9 (≥22/<22) | 2.125 | 1.580-2.858 | <0.001 | 2.677 | 1.461-4.905 | 0.001 |  |
| Tumor size (>5/≤5) | 1.218 | 0.956-1.553 | 0.110 | 1.215 | 0.778-1.897 | 0.392 |  |
| Solitary tumor | 0.608 | 0.473-0.783 | <0.001 | 0.547 | 0.350-0.853 | 0.008 |  |
| Differentiation |  |  |  |  |  |  |  |
| Well | Ref | Ref |  | Ref | Ref |  |  |
| Moderate | 2.231 | 0.829-6.005 | 0.112 | 3.427 | 0.475-24.739 | 0.222 |  |
| Poor | 3.140 | 1.133-8.703 | 0.028 | 5.985 | 0.788-45.453 | 0.084 |  |
| Macrovascular invasion | 1.126 | 0.849-1.493 | 0.409 | 1.005 | 0.635-1.589 | 0.984 |  |
| Microvascular invasion | 1.772 | 1.261-2.491 | 0.001 | 2.391 | 1.252-4.566 | 0.008 |  |
| Cirrhosis | 1.360 | 1.054-1.754 | 0.018 | 1.363 | 0.759-2.447 | 0.300 |  |

ALBI, albumin-bilirubin; AAPR, albumin-to-alkaline phosphatase ratio; M, male; F, female; ALB, albumin; TBIL, total bilirubin; ALP, alkaline phosphatase; CA19-9, carbohydrate antigen 19-9; HR, hazard ratio; CI, confidence interval; Ref, reference.

Supplementary table 3. Multivariate analysis showing independent prognostic factors for overall survival in the derivation cohort.

| Variables | Multivariate | | | |
| --- | --- | --- | --- | --- |
|  | HR | 95% CI | *P* |  |
| Gender (F/M) | 0.815 | 0.631-1.053 | 0.118 |  |
| Hepatolithiasis | 1.170 | 0.839-1.631 | 0.356 |  |
| Child-Pugh (5/6) | 1.282 | 0.380-4.323 | 0.689 |  |
| Ascites (+/-) | 1.180 | 0.788-1.767 | 0.423 |  |
| ALB | 0.964 | 0.934-0.995 | 0.023 |  |
| TBIL | 0.985 | 0.965-1.005 | 0.134 |  |
| ALP | 1.002 | 1.000-1.003 | 0.008 |  |
| CA19-9 (≥22/<22) | 2.235 | 1.635-3.054 | <0.001 |  |
| Tumor size (>5/≤5) | 1.005 | 0.956-1.056 | 0.838 |  |
| Solitary tumor | 0.556 | 0.417-0.742 | <0.001 |  |
| Differentiation |  |  |  |  |
| Well | Ref | Ref |  |  |
| Moderate | 1.953 | 0.618-6.171 | 0.254 |  |
| Poor | 2.839 | 0.811-9.343 | 0.082 |  |
| Microvascular invasion | 1.586 | 1.106-2.272 | 0.012 |  |
| Cirrhosis | 1.525 | 1.145-2.031 | 0.004 |  |

M, male; F, female; ALB, albumin; TBIL, total bilirubin; ALP, alkaline phosphatase; CA19-9, carbohydrate antigen19-9; HR, hazard ratio; CI, confidence interval; Ref, reference. ALB, ALP and TBIL were simultaneously put in, whereas ALBI and AAPR were excluded.

Supplementary table 4. Short-term outcomes of ICC patients stratified by ALBI and AAPR grade.

| Variables | ALBI | | | AAPR | | |
| --- | --- | --- | --- | --- | --- | --- |
|  | Grade 1 | Grade 2 | P value | >0.348 | ≤0.348 | P value |
| Derivation cohort | | | | | | |
| Patient number | 387 | 90 |  | 293 | 184 |  |
| Operative time, [min, mean (SD)] | 212 (71) | 221 (63) | 0.269 | 225 (58) | 215 (55) | 0.252 |
| Estimated blood loss, [ml, mean (SD)] | 326 (142) | 341 (126) | 0.357 | 332 (102) | 347 (135) | 0.169 |
| Intraoperative transfusion, n (%) | 35 (9.1%) | 10 (11.1%) | 0.565 | 24 (8.2%) | 21 (11.4%) | 0.265 |
| Hospitalization, [day, mean (SD)] | 5.4 (2.3) | 5.8 (2.1) | 0.132 | 5.5 (2.4) | 5.7 (1.9) | 0.339 |
| Postoperative complications, n (%) | 70 (18.1%) | 18 (20%) | 0.704 | 46 (15.7%) | 42 (22.8%) | 0.078 |
| Major complications, n (%) | 8 (2.1%) | 4 (4.4%) | 0.201 | 7 (2.4%) | 5 (2.7%) | 0.826 |
| Validation cohort | | | | | | |
| Patient number | 103 | 40 |  | 76 | 67 |  |
| Operative time, [min, mean (SD)] | 234 (71) | 249 (65) | 0.248 | 238 (81) | 252 (68) | 0.268 |
| Estimated blood loss, [ml, mean (SD)] | 356 (163) | 397 (222) | 0.227 | 361 (179) | 411 (215) | 0.132 |
| Intraoperative transfusion, n (%) | 10 (9.7%) | 5 (12.5%) | 0.644 | 6 (7.9%) | 9 (13.4%) | 0.308 |
| Hospitalization, [day, mean (SD)] | 6.3 (1.9) | 6.9 (2.1) | 0.102 | 6.2 (2.1) | 6.8 (2.3) | 0.105 |
| Postoperative complications, n (%) | 16 (15.5%) | 10 (25%) | 0.233 | 15 (19.7%) | 11 (16.4%) | 0.642 |
| Major complications, n (%) | 4 (3.9%) | 3 (7.5%) | 0.381 | 3 (3.9%) | 4 (5.9%) | 0.585 |

ALBI, albumin-bilirubin; AAPR, albumin-to-alkaline phosphatase ratio; major complications were classified according to Clavien-Dindo classification (grade III or higher).

**Supplementary figure legends**

**Supplementary figure 1**. Kaplan-Meier curves showing overall survival of 477 surgically treated ICC patients in the derivation cohort stratified by: Child-Pugh score (A), CA19-9 (B), cirrhosis (C), tumor number (D), tumor differentiation (E) and microvascular invasion (F).

**Supplementary figure 2**. Kaplan-Meier curves showing overall survival of 143 surgically treated ICC patients in the validation cohort stratified by: Child-Pugh score (A), CA19-9 (B), cirrhosis (C), tumor number (D), tumor differentiation (E) and microvascular invasion (F).


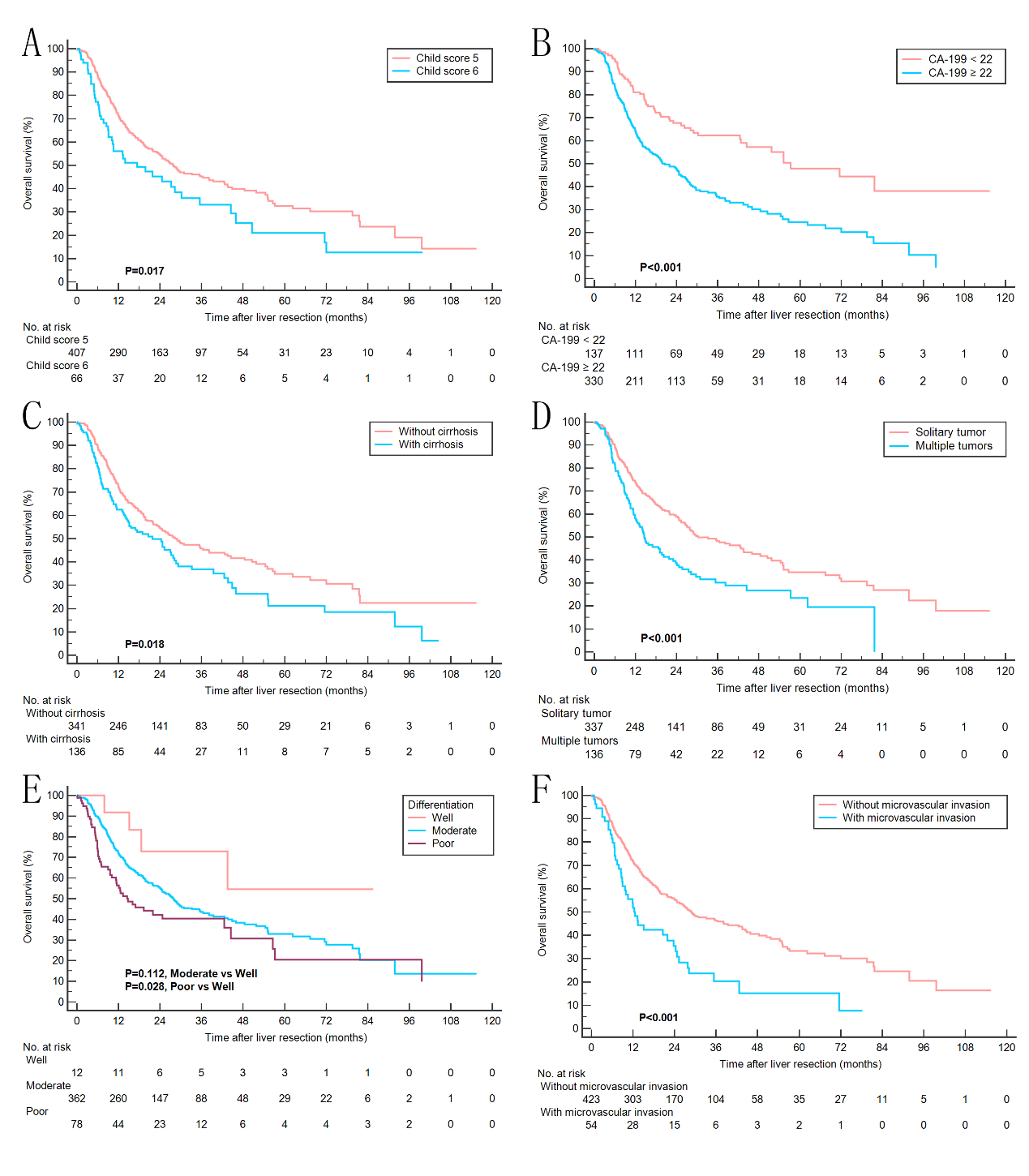


Supplementary figure 1


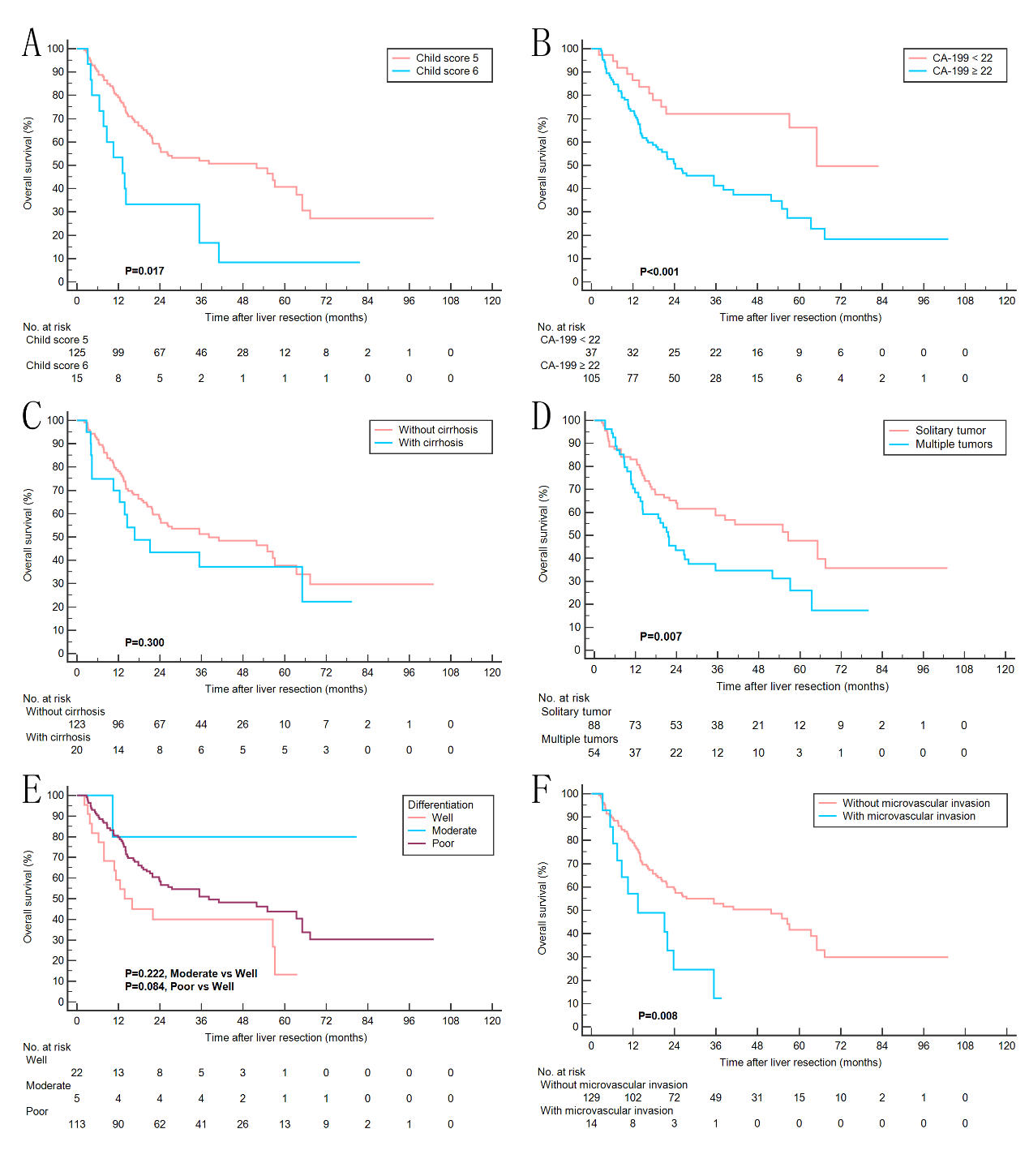


Supplementary figure 2
